# An electromyographic study of abdominal muscle activity in children with spastic cerebral palsy

**DOI:** 10.4102/sajp.v73i1.341

**Published:** 2017-10-20

**Authors:** Saviour K. Adjenti, Graham Louw, Jennifer Jelsma, Marianne Unger

**Affiliations:** 1 Department of Anatomy, School of Biomedical & Allied Health Sciences, College of Health Sciences, University of Ghana, Ghana; 2 Division of Clinical Anatomy & Biological Anthropology, Department of Human Biology, Faculty of Health Sciences, University of Cape Town, South Africa; 3 Division of Physiotherapy, Department of Health & Rehabilitation Sciences, University of Cape Town, South Africa; 4 Division of Physiotherapy, Faculty of Medicine and Health Sciences, Stellenbosch University, South Africa

## Abstract

**Background:**

Inadequate knowledge in the recruitment patterns of abdominal muscles in individuals with spastic-type cerebral palsy (STCP).

**Objectives:**

To determine whether there is any difference between the neuromuscular activity (activation pattern) of the abdominal muscles in children with STCP and those of their typically developing (TD) peers.

**Method:**

The NORAXAN^®^ electromyography (EMG) was used to monitor the neuromuscular activity in abdominal muscles of individuals with STCP (*n* = 63), and the results were compared with the findings from age-matched TD individuals (*n* = 82).

**Results:**

EMG frequencies were recorded during rest and during active states and compared using repeated measures ANOVA. Spearman’s rank order correlation was used to explore relationships between age, body mass index and abdominal muscle activity. With the exception of the rectus abdominis (RA) muscle, the pattern of neuromuscular activity in children with STCP differs significantly from that of their TD peers. Three of the muscles – external oblique abdominis (EO), internal oblique abdominis (IO) and RA – in both groups showed significant changes (*p* < 0.001) in the frequency of EMG activity between the resting and active states. An elevated EMG activity at rest in the EO and IO was recorded in the STCP group, whereas the RA during resting and active stages showed similar results to TD individuals.

**Conclusion:**

The findings from this study suggest that the RA could be targeted during rehabilitation regimens; however, the force generated by this muscle may not be sufficient for the maintenance of trunk stability without optimal support from the EO and IO muscles.

## Background

In order to maintain the necessary equilibrium of stability and mobility of the spine, recruitment of trunk muscles has to be adequately organised and coordinated (Tucker & Hodges 2010). In healthy individuals, the global system of which the abdominal muscles form part is known to recruit fibres at different intensities to produce movements (Vasseljen et al. 2009). Similarly, these muscles need to contract appropriately, eccentrically and isometrically, to contribute to the stability of the trunk (Hagberg et al. 2001; Tucker & Hodges 2010). Muscle weakness and morphological disorientation of the trunk are the typical physical signs commonly associated with low back pain (Tucker & Hodges 2010). These are also said to resemble the physical characteristics of individuals with spastic-type cerebral palsy (STCP) (Rosenbaum et al. 2007).

In children with cerebral palsy (CP), hypertonia and muscle weakness are reported to be the common impairments contributing in varying degrees to motor dysfunction seen in these children (Rosenbaum et al. 2007). These impairments are also found in the trunk musculature, and ascertaining their contribution to postural control, including pelvic stability, is essential in planning treatment for these children (Damiano & Moreau 2008). Hypertonia in the trunk, however, is not just because of brain lesion but can be attributed to being a compensatory strategy because of peripheral muscle weakness (Hagberg et al. 2001). Hypertonia also causes transformations in muscle morphology (Chapman et al. 2008), which further impacts the ability of these muscles to contract. The relationship between the abdominal muscle strength or weakness and firing patterns of these muscles, however, is not well described in CP and how these differ from age-matched peers of the typically developing (TD) group is not fully understood. Because the abdominal muscles have been considered as forming part of the global system, and therefore are trunk stabilisers, a comprehensive knowledge of their recruitment patterns may be useful during rehabilitation regimens for individuals with STCP.

There are, however, limited studies that aim to describe recruitment strategies of the trunk muscles in CP, but these are predominantly inferred from observational analysis (Tucker & Hodges 2010; Vasseljen et al. 2009). Prosser et al. (2010) used electromyography (EMG) to study activity in trunk and hip muscles in early walkers with and without STCP. From this study, the mean EMG frequencies in children with STCP in all the muscles measured were significantly elevated. According to these authors, the high frequencies recorded during gait could be attributed to muscle fatigue and not necessarily an indication of force generation. There were also reports of a correlation between abnormal gait, altered recruitment patterns and trunk musculature from the study of Prosser and co-workers. Some of these investigations with counterintuitive results serve as the basis of motivation for this study, especially when no further studies on children with STCP and recruitment patterns of the trunk musculature could be found. Our motivation also comes from reports that indicate that even in healthy individuals the recruitment pattern of the global muscles is characterised with varied intensities (Vasseljen et al. 2009).

Our study, therefore, aimed to investigate and compare the neuromuscular activity of the anterior trunk muscles (trunk stabilisers) in children with STCP with their age-matched TD peers. Measurements at rest and during activity were recorded using surface electromyography (sEMG). We hypothesised that selected EMG measurements would reflect the degree of contractile response in the abdominal muscles in individuals with STCP and that this would differ from that recorded in children with TD. Relationships between age and body mass index (BMI) and abdominal muscle activity were also explored.

## Materials and methods

A descriptive correlational study design was used in that the EMG recordings of abdominal muscle activity in children with STCP and those with age-matched TD peers were compared. Ethical approval was obtained from the Human Research Ethics Committee of the University of Cape Town (HREC REF: 490/2011). Permission to recruit the learners for the study was obtained from The Western Cape Department of Education for the participating schools in the Cape Town Metropole (REF: 2011202-0028). Recruitment was done in three schools for children with special education needs and two randomly selected public mainstream schools located within the vicinity of the special schools. All children who complied with the inclusion criteria and whose parents or legal guardian gave written informed consent were invited to participate.

### Participants

A final group of 63 children aged between 7 and 16 years with STCP was compared to a group of 82 TD individuals. The two groups were not matched for any demographic or anthropometric variables. Differences were explored and, where appropriate, factored into the statistical analysis. For both groups, any child was excluded if he or she had any surgical procedure involving the anterior abdominal wall in the last 6 months before the start of the study. Children with STCP had to be ambulant (i.e. level I-IV according the GMFCS [Palisano et al. 2000]). Any individual with STCP at level V was excluded because they were unable to perform the test manoeuvre. Learners who had undergone any medical treatment that would have impacted on muscle function (e.g. botulinum toxin injection, casting and surgical intervention such as dorsal rhizotomy and baclofen pump placement) less than 6 months before the study were also excluded.

### Instrumentation

The NORAXON^®^ sEMG was used in this study as EMG is considered a more objective method for describing muscle activation patterns. sEMG has been shown to be a valid and reliable method for detecting muscle activity in CP, and the limitations on the use of surface electrodes have been well described by Hlustik et al. (2004). Fine wire electrodes are considered the gold standard, but as this method is invasive and potentially painful, it is considered unethical to use in the paediatric population (Hermens et al. 2000).

Each recording channel on the equipment is designed to detect activity from one muscle site and is composed of two active electrodes and a reference one. Surface EMG uses disc-coated silver-silver chloride electrodes that can detect algebraic sums of voltages associated with muscle action potential within their pick zones. The electrodes are about 0.5–1.0 cm in their wider diameter. The difference in electrical charges between each active electrode and the reference one provides input to a differential amplifier with impedance of a common mode rejection ratio (CMMR) between 90 and 140 dB. There are filters as part of the internal design of this NORAXON^®^ sEMG equipment, which allow frequencies related to a muscle activity but reject those frequencies that are associated with electromagnetic noise. According to the literature, the evaluation of EMG by means of amplitude is influenced by many factors: anthropometrics, precise electrode placement and overlying tissues in the body (Moreau, Holthaus & Marlow 2013; Prosser et al. 2010). Additionally, reports indicate that the analysis of EMG amplitude in millivolt units could only be useful in reflecting the neuromuscular activity in a single muscle or a group of muscles, whereas the evaluation of neuromuscular activity in the form of rate or frequency measured in Hertz (Hz) units is the preferred choice for comparing multiple muscles across individuals (Prosser et al. 2010). Based on this evidence, we chose the latter method because our evaluation involved comparisons of the activities of all four abdominal muscles in two different groups. In this study, the signals obtained were viewed in their ‘raw’ plus or minus form and then converted to a unidirectional signal in which the ‘plus-minus’ waveform was rectified.

### Procedure

The study aimed to record EMG at rest and during active state. Limb movement is reported to be accompanied by a concomitant contraction of the abdominal muscles (Vasseljen et al. 2009), and thus, for the purpose of this study, it was deemed appropriate to use head and leg movements with the participant in supine position to record isometric abdominal muscle activity using sEMG. For this study, the active electrodes were spaced with their centres about 2 cm apart on three of the four abdominal muscles, namely rectus abdominis (RA), internal oblique (IO) and external oblique (EO) muscles. The transversus abdominis (TrA) was practically impossible to access by virtue of the deep position of this muscle with respect to the exterior of the anterior abdominal wall.

Prior to the collection of the data, participants were trained to reach paced activities within 1 min with regard to tasks to be accomplished during active or contracted phases (head up with chin tucked in and lower limb movements). For the latter, the more affected limb was identified by a neurodevelopmental therapist for the participant in the STCP group. In the supine position on a plinth with the arms resting along the sides of the body, the children were asked to flex the hip and knee and bend up as far as possible. For the head and neck movements, participants were instructed to tuck in their chins and then lift their heads to their chests. All of these movements were performed under the supervision of their physiotherapist.

Testing was officially initiated (usually a day or two after training) when participants appeared to be confident and consistent with executing the tasks. All the silver-silver chloride electrodes came prepared with a creamed electrode gel that makes them self-adhesive. However, electrode placements were reinforced with adhesive sellotape. The electrode sites were gently abraded with fine grain sand paper and cleaned with isopropyl alcohol. The placement of the electrodes was in accordance with standard procedures, (Hermens et al. 2000; Ng, Kippers & Richardson 1998).

For the RA, the active electrodes were placed 3 cm above the umbilicus and 3 cm away from the median plane always on the right side but if unilaterally affected as in the case of right hemiplegia, then placement of this electrode was done on the left side. The ground electrode was placed at the level of the 12^th^ thoracic spinous process (T12) for those participants with spastic diplegia and quadriplegia, whereas this electrode was placed on the patella of the non-affected limb for participants from the hemiplegic and TD groups. For the EO EMG readings, the active electrodes were placed midway between anterior superior iliac spine (ASIS) and the lowest point on the subcostal angle in the mid-axillary line always on the right side, but if unilaterally affected, then on the left. Alternatively, the more lateral electrode was placed at the most lateral point in the mid-axillary line on the trans-umbilical plane on the affected side. The EMG recordings for IO were taken with active electrodes at a point midway between the ASIS and pubic tubercle, about 2 cm above the inguinal ligament in the mid-clavicular line always on the right side, but if unilaterally affected, then on the left. In the case of children from the TD groups, the active electrodes were placed at these anatomical sites as described for the above but always on the right side.

The sEMG were sampled at 1000 Hz and saved on a personal computer for further analysis with a custom made DELL/NORAXAM programme. Based on a trial test, patterns of sEMG activity in the participants were found to be variable across 10 trials, and therefore, the tasks were limited to a maximum of 5 after which the mean scores of 3 consistent patterns were recorded as the sEMG score for a particular muscle. The raw sEMG signals were first amplified 300 times by pre-amplified electrodes by default and then 4 times with the computer analysis. The signals were then filtered with 10–1000 Hz band per filter. All data were then filtered with a second low-pass filter at 16 Hz. A muscle onset of activity was defined as the point when sEMG recording exceeded the baseline by two standard deviations for greater than 25 ms and the software marks this point as the EMG traces. These were then visually inspected by the principal investigator (PI) and the research assistant independently ensured that subsequent outbursts or values/traces obtained could be compared to determine inter-tester (between PI and research assistant) and intra-tester reliability (within day activity of PI) (see Tables 1–3 for validity and reliability results).

**TABLE 1:** Comparison of demographic data between the two groups.

Variables	Mean STCP	SD STCP	Mean TD	SD TD	*p*
Age (years)	11.9	2.92	11.0	3.04	0.087
Height (cm)	139.2	16.04	143.3	17.13	0.213
Weight (kg)	39.7	10.28	38.7	12.38	0.457
BMI (kgm^−2^)	20.1	2.16	18.4	2.62	< 0.001

*Source*: Authors’ own work

SD, standard deviation; TD, typically developing; STCP, spastic-type cerebral palsy; BMI, body mass index.

**TABLE 2:** Descriptive statistics for electromyograph measurement (raw scores) during resting and active stages for both groups of participants (STCP: *N* = 63; TD: *N* = 82).

Diagnosis	Muscle type and state	Mean EMG (Hz)	SD	Diff	SD of Diff	95% CI of Diff	*p*
STCP	EO R	85.0	2.3				0.001
	EO Ac	123.1	2.5	38.1	2.1	37.6–38.6	
TD	EO R	11.8	1.6				
	EO Ac	107.3	1.8	95.5	1.5	95.1–98.0	
STCP	IO R	89.9	4.0				0.001
	IO Ac	127.5	3.8	37.7	2.8	37.0–38.4	
TD	IO R	11.6	1.6				
	IO Ac	110.8	2.0	99.2	1.7	98.6–99.8	
STCP	RA R	12.2	1.4				0.074
	RA Ac	97.3	1.8	85.1	1.2	84.8–85.4	
TD	RA R	11.4	1.4				
	RA Ac	97.0	1.8	85.6	1.3	85.3–88.3	

*Source*: Authors’ own work

EMG, electromyography; R, resting state; Ac, active state; SD, standard deviation; Diff, differences in EMG scores between active and resting states; CI, confidence interval; STCP, spastic-type cerebral palsy; TD, typically developing; RA, rectus abdominis; EO, external oblique abdominis.

**TABLE 3:** Spearman’s correlation between the electromyograph activity of the abdominal muscles during the resting and active stages as well as between age and body mass index of participants.

Variables	STCP – 63		TD – 82	
	Spearman	*p*	Spearman	*p*
Resting stage – Age versus EMG				
EO R EMG (Hz)	−0.71	0.001	−0.84	0.001
IO R EMG (Hz)	−0.73	0.001	−0.88	0.001
RA R EMG (Hz)	−0.61	0.001	−0.89	0.001
Active stage – Age versus EMG				
EO Ac EMG (Hz)	−0.26	0.036	−0.76	0.001
IO Ac EMG (Hz)	−0.32	0.012	−0.74	0.001
RA Ac EMG (Hz)	−0.64	0.001	−0.81	0.001
Resting stage – BMI versus EMG				
BMI and EO R EMG (Hz)	−0.04	0.764	−0.36	0.001
BMI and IO R EMG (Hz)	−0.06	0.653	−0.36	0.001
BMI and RA R EMG (Hz)	0.00	0.974	−0.31	0.004
Active stage – BMI versus EMG				
BMI and EO Ac EMG (Hz)	−0.11	0.400	−0.27	0.015
BMI and IO Ac EMG (Hz)	−0.03	0.793	−0.34	0.002
BMI and RA Ac EMG (Hz)	−0.07	0.578	−0.39	0.001

*Source*: Authors’ own work

R, resting state; Ac, active state; STCP, spastic-type cerebral palsy; TD, typically developing; RA, rectus abdominis; EO, external oblique abdominis; EMG, electromyography; Hz, Hertz; BMI, body mass index.

### Statistical analysis

STATISTICA software package, version 11 (2012), was used to analyse the data. Descriptive statistics were presented. The Shapiro-Wilk test indicated that most data sets were normally distributed. Spearman’s rank order correlation was calculated between age, BMI and muscle activity. Although age was shown to consistently predict EMG scores, results using data normalised for age did not differ from analysis using raw data and are therefore not presented as such in this article. Repeated measures ANOVA were used to compare resting and active state sEMG scores both within and between groups. The level of significance for all statistical tests was set at 0.05.

## Results

### Description of the sample

Table 1 gives a description and comparison of the age, height, weight and BMI of the individuals who took part in this study. Of the 63 participants with STCP, 34 were classified as level I, 11 as level II, 8 as level III and 10 as level IV. There were no significant differences in age (*p* = 0.09), height (*p* = 0.21) or weight (*p* = 0.46) between the two groups. The children with STCP were both shorter and heavier than the children in the TD group and therefore also presented with a significantly greater BMI (*p* < 0.001).

### Muscle activity during rest and activity

The results shown in Table 2 indicate that for all three abdominal muscles even at rest, the scores were well above zero mark in both groups of children. For the EO and IO muscles, the resting scores were, however, significantly higher (*p* < 0.001) in individuals with STCP compared to those recorded in TD children. No difference was seen between the two groups for the RA at rest.

During the active state, the sEMG scores were again higher in the STCP group compared to the TD group for the IO and EO muscles. Again no difference was seen for the RA muscle between the two groups.

For all three muscle groups, there was a significant increase in EMG activity from resting to active states with *p* < 0.001. The difference in EMG frequency recording from rest to active states was significantly higher in TD children when compared to children with STCP for the EO and IO muscles with *p* < 0.001. No difference was seen in the RA muscles between the two groups (*p* = 0.074).

### Correlation of electromyography with age

Relationships between age, BMI and muscle activity were explored and are shown in Table 3. The Spearman’s correlation results showed that age and, to a lesser extent, BMI are related to abdominal muscle activity in both groups. At rest, a negative correlation exists between age and activity recorded in that activity decreases with age. In TD children, this was also true for abdominal activity recorded during activity. For children with STCP, this was only true for the RA muscle. No relationship seems to exist for the other two muscles (Table 3). The BMI and EMG activity in the TD group showed somewhat weak negative correlation, whereas there was no correlation between the EMG activity and BMI with respect to the STCP group at either stage.

**FIGURE 1:**
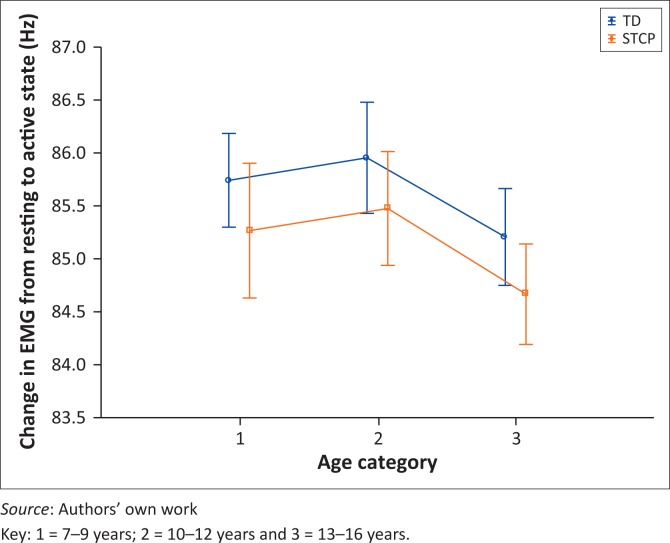
Graphical representation of changes in rectus abdominis thickness from resting to active state against categorised ages in years for both groups (*N* = 145).

Figure 1 shows that the smallest change in recorded EMG activity in the RA can be seen in the older children, and the greatest change in the 10–12-year-old grouping, possibly suggesting that in older children, the RA is recruited less as they get older. Similar change patterns were noticed, however, for the other muscle groups.

Figure 1 also highlights that the RA muscle of the STCP group seems least affected by tone both at rest and during activity.

## Discussion

This study has shown that similarities and differences exist in the EMG activity recorded for the abdominal muscles in children with STCP and their age-matched TD peers. Although the RA of the children with STCP showed similar patterns of recruitment to those of TD children, activity in the other two abdominal muscles recorded in this study differed significantly both at rest and during contraction. Despite the demographic data showing that the individuals in the STCP group were heavier than their age-matched TD counterparts (refer to Table 1), no direct link could be found between the EMG scores and BMI of these individuals with STCP. TD In children abdominal muscle activity seems to decrease during the same head or leg lift activity as they get older. We discuss the possible implications of these findings below.

The sEMG scores recorded during the resting state were above absolute zero for both groups of children. With respect to the individuals with STCP, our results showed that these scores were significantly elevated, suggestive of hypertonia (Rosenbaum et al. 2007). It appears almost certain that despite practice sessions, being unfamiliar with the testing protocol may have increased anxiety levels in the participants accounting partially for these initial seemingly exaggerated (above the absolute zero mark) scores during the resting stage in both groups. The exact stimulus or mechanism by which the EO and IO muscles recruit in order to sustain the increased tone while at rest remains unclear. Although the measurements recorded during the resting phase especially in the STCP group could be because of increased and uninhibited activity of motor neurons in these muscles, all the measurements were positive, that is, above the zero mark for participants in both groups. For the EMG activity at the resting stage to be above the absolute zero mark is worthy of note against the backdrop that participants were fully resting on a plinth with any possible muscular activity counteracted by being supported in a supine position. These resting stage scores especially in the TD group were not totally unexpected because resting tone is what enables or determines the quality of the physical activity of skeletal muscles. Our results, therefore, are in support of the conclusion of Damiano and Moreau (2008) that any healthy muscle at rest exhibits a low EMG frequency, close to but never a score of zero.

This study appears to suggest that during the active stage, EO and IO muscles in children with STCP showed an inability to synchronise the rate of activation of all the already activated motor units. The small difference found between the two groups in terms of changes in EMG activity during the resting and active stages is most likely because of the elevated resting stage measurements seen in the STCP group. The changes in EMG activity between the active and resting stages in the IO and EO muscles could be explained on the basis of these muscles being in a state of fatigue prior to the demand for work output (active stage) which would negatively affect their ability to (recruit fibres) contract fully. Our findings therefore are in support of other studies that report the existence of a correlation between over-contraction and fatigue in muscles and low or poor EMG activity (Dario, Merletti & Enoka 2004; Suzuki et al. 2002). The EMG scores in both groups during the active stages, however, are similar, which may imply that at the structural level, the numbers of contractile elements in each of these muscles from the STCP and TD groups are relatively of approximate proportion (Moreau et al. 2013).

Some other studies have shown that the magnitude of the differences in neuromuscular activity between the resting and active stages, measured in Hertz units (change in EMG frequency), is indicative of the strength of a given muscle (Chapman et al. 2008; Fosang & Baker 2006). In this study, there were significant increases in EMG frequencies from resting to active stages in both groups. The difference in the magnitude of these changes in EMG activity between the active and resting stages between the two groups might explain the differences seen in the performance of the abdominal muscles in the maintenance of posture. The changes shown in the STCP group for the oblique muscles were smaller than the TD group. This observation seems suggestive that the EO and IO muscles in individuals with STCP are likely to be weaker muscles than the RA muscle. Consequently, on the basis of these differences between the RA and the oblique muscles in the two groups, the concept of poor synchronisation between the depolarisation and repolarisation phases of the motor-end-plates may also explain the trend in the sEMG scores as seen above. The occurrence or the absence of appropriate physiological events might represent the concept of ‘fatigue’ in muscle groups with increased tone (Ohata et al. 2008; Prosser et al. 2010). With the EO and IO muscles in the STCP group during the active stage, it is not clear whether new motor units were recruited in addition to the already activated motor units (to maintain tone). From the literature, both mechanisms, constant activation and the recruitment of additional motor units, have been associated with spastic muscles (Hodges et al. 2005; Tucker & Hodges 2010).

The EMG frequency is reported to correspond to recruitment patterns but is not necessarily related to performance levels of a muscle (Gough & Shortland 2012; Prosser et al. 2010). Had the EMG activity in this study, as measured in Hertz units (frequency) during the active stage in the STCP group, remained constant or even decreased below the resting stage scores, it could have been interpreted as inability to recruit additional (new) motor end-plates for the specific tasks. From our results as presented above, it is prudent to assume that with the EO and IO muscles in the STCP group, little or no force is being generated towards moving or stabilising the trunk or bony pelvis. This is likely to be the case because the state of electrical activity in the oblique muscles for the children with STCP is characterised by an initial (resting stage) high EMG score that renders the net result during the active stage ineffective in relation to performance or force generation.

In contrast to EO and IO abdominal muscles, the RA muscle in the STCP group showed electrical activity typical of TD individuals. It suffices to conclude that if high EMG scores at rest signify spasticity or increased tone or muscle weakness, then neither of these characterised the RA muscle in individuals with STCP. The RA in the STCP group as well as the muscles in the TD group appear to be more relaxed with some resting tone and therefore able to activate sufficient motor units during activity. Low muscle tone during the resting stage indicates healthy muscle activity (Damiano & Moreau 2008; Gough & Shortland 2012) because any healthy muscle at rest exhibits a low EMG frequency but never a score of zero. The low EMG scores of the RA at rest may imply that during contraction all the depolarisation of the motor units becomes effective in reflecting the typical state of the electrical activity of this muscle. Therefore by inference, the RA muscle in individuals with STCP is the least affected by spasticity. This muscle could, therefore, be targeted during rehabilitation procedures to provide the necessary contraction required to stabilise the trunk, since it functionally belongs to the global system of trunk muscles (Damiano et al. 2000; Vasseljen et al. 2009) – capable of effecting movement at the bony pelvis. We suggest, however, that attention be given to the total core muscles during physical training as over reliance on the RA leads to trunk rigidity and low back pain in the long term (Hodges et al. 2005).

Alternatively, the difference in the amount of change in EMG activity between the oblique and RA muscles in the STCP group with regard to the provision of trunk stability may possibly be explained using the concept of agonistic and antagonistic behaviours of the skeletal muscles. It is possible that, with the alteration of the neuromuscular system as associated with STCP, co-contraction takes place between the RA and the remaining abdominal muscles, similar to muscles acting as agonists and antagonists (Damiano et al. 2000; Damiano & Moreau 2008). The concept of co-contraction for any reason in agonistic or antagonistic muscles has been shown to increase joint stiffness that makes movement strenuous (Moreau, Teefey & Damiano 2010; Moseley & Hodges 2005). This increased stiffness of joints and difficulty of movement is that which has been observed with a pelvic tilt in individuals with STCP (assuming these muscles, the rectus and obliquus abdominis, are behaving as agonists and antagonists); then the levels of force production could only be maintained if the force of agonist increased concurrently with antagonistic restraint. The result of this type of action between two physiologically different muscles is that the joint could become incrementally stiffer, a feature that is commonly evident at the pelvis in individuals with STCP (Damiano & Moreau 2008). These differences in the changes to muscle thickness and EMG activity between the EO and IO muscles on one hand and the RA muscle on the other in individuals with STCP mean that the possibility of co-contraction amongst abdominal muscles must not be overlooked during rehabilitation and management of these individuals.

### Limitations of the study

The TrA muscle is also considered as an important trunk muscle alongside the IO and, therefore, the electrical activity from the TrA muscle would have been useful in establishing its role with respect to trunk stability and maintenance of posture. However, owing to the use of sEMG in this study, any inference of TrA has been excluded. Participants could not be restricted from physical activity prior to testing sessions which may have influenced the resting state sEMG measurements.

## Conclusion and recommendation

The EO and IO muscles in the STCP group have higher sEMG scores at rest than their TD peers, suggesting that these muscles are actively contracting even at rest. The RA muscle in individuals with STCP recorded sEMG scores comparable to those of TD individuals. The sEMG results for the RA muscle in individuals with STCP suggest that the RA muscle in individuals with STCP can contract optimally, therefore appear unaffected or least affected by the condition of STCP. With the exception of the RA muscle, the neuromuscular activities (recruitment patterns) of individuals with STCP differ significantly from those of TD individuals.

There is a need for further research as to the underlying causes of the increased tone in the EO and IO muscles at rest as reflected in the elevated sEMG scores (above the zero score mark), especially in the STCP group: whether neurological damage or simply a physiological adaptation was beyond this study and therefore requires further investigation, especially why the RA muscle appears to be unaffected. This study has implication for clinical practice. In terms of therapy, it would appear necessary to attempt to reduce the tone of the EO and IO muscles before attempting to recruit and strengthen these oblique muscles during functional activities. This may be either direct or in cases where the over recruitment is a compensatory strategy, balance between agonists and antagonists should be sought in order to reduce over reliance on any one group for stability. It is also necessary to attempt to selectively train and strengthen the EO and IO muscles to reduce the over reliance on the RA muscle resulting in a rigid trunk. It may be worth looking into the mobility of the pelvis and how this influences recruitment of the RA muscle.
